# Maternal Morbidity and Medically Assisted Reproduction Treatment Types

**DOI:** 10.1097/AOG.0000000000005808

**Published:** 2024-12-19

**Authors:** Alina Pelikh, Ken R. Smith, Mikko Myrskylä, Michelle P. Debbink, Alice Goisis

**Affiliations:** Centre for Longitudinal Studies, Social Research Institute, University College London, London, United Kingdom; Population Science, Huntsman Cancer Institute, the Department of Family and Consumer Studies, the Department of Obstetrics and Gynecology, and the Spencer Fox Eccles School of Medicine, University of Utah, Salt Lake City; Max Planck Institute for Demographic Research, Rostock, Germany; the Helsinki Institute for Demography and Population Health, University of Helsinki, Helsinki, Finland; and the Max Planck – University of Helsinki Center for Social Inequalities in Population Health, Rostock, Germany and Helsinki, Finland.

## Abstract

**OBJECTIVE:**

To compare odds of maternal morbidity by mode of becoming pregnant and type of medically assisted reproduction treatments: fertility-enhancing drugs, intrauterine insemination (IUI), and assisted reproductive technology (ART) with autologous or donor oocytes.

**METHODS:**

Birth certificates were used to study maternal morbidity among the birthing population in Utah between 2009 and 2017 (N=469,919 deliveries); 22,543 pregnancies occurred through medically assisted reproduction (4.8%). Maternal morbidity was identified as a binary variable, indicating the presence of any of the following: blood transfusion, unplanned operating room procedure, admission to intensive care unit, eclampsia, unplanned hysterectomy, and ruptured uterus. Using logistic regression, we assessed maternal morbidity among medically assisted reproduction pregnancies (overall and by type of treatment) compared with unassisted pregnancies in the overall sample before and after adjustment for individual sociodemographics (age at birth, family structure, level of education, Hispanic origin, parity), and pre-existing comorbidities (ie, chronic hypertension, heart disease, asthma), multifetal gestation, and obstetric comorbidities (ie, placenta previa, placental abruption, cesarean delivery).

**RESULTS:**

Individuals becoming pregnant through medically assisted reproduction had higher risk of maternal morbidity, with odds ratios (ORs) increasing as medically assisted reproduction treatments become more invasive. Associations were largely attenuated when accounting for multifetal gestation and further reduced after controlling for obstetric comorbidities. However, ART with autologous oocytes (OR 1.46, 95% CI, 1.20–1.78) maintained higher coefficients compared with unassisted pregnancies. In models including only singletons, after controlling for obstetric comorbidities, the OR differences in maternal morbidity between all medically assisted reproduction groups and unassisted pregnancies were no longer statistically significant.

**CONCLUSION:**

More invasive medically assisted reproduction treatments (ART and IUI) are associated with higher odds of maternal morbidity, whereas less invasive treatments are not. This relationship is partially explained by higher prevalence of multifetal gestation and obstetric comorbidities in people undergoing more invasive treatment, but the persistent association suggests subfertility itself may contribute to maternal morbidity.

## Abstract 2

More invasive medically assisted reproduction treatments are associated with higher odds of maternal morbidity, whereas less invasive treatments show similar odds of maternal morbidity as becoming pregnant unassisted.

The increasing use of medically assisted reproduction (ie, in vitro fertilization, intracytoplasmic sperm injection [ICSI], ovulation induction) requires better understanding of its effects on maternal and child health. Although research has focused on pediatric outcomes,^1–4^ less attention has been given to maternal morbidity. Studies show mixed findings, with some reporting increased risks among individuals conceiving through medically assisted reproduction^5–8^ and others reporting elevated risk only in specific high-risk subgroups such individuals with multifetal gestation or pre-existing health conditions.^9–12^ The complex, interconnected factors underlying these associations, which include subfertility and pre-existing comorbidities, sociodemographic characteristics and obstetric complications,^13–17^ make it difficult to assess whether medically assisted reproduction treatment itself increases maternal morbidity risk. The choice of medically assisted reproduction procedures depends on factors such as infertility duration, diagnosis, availability, and cost,^18,19^ typically progressing from less to more invasive treatments. Limited large-scale data have restricted studies investigating maternal morbidity differences by medically assisted reproduction treatment types. Existing studies show higher maternal morbidity risks for more invasive treatments (in vitro fertilization or ICSI) compared with becoming pregnant unassisted, with mixed findings for less invasive treatment.^9–12^

The primary objective of this study was to compare odds of maternal morbidity by mode of becoming pregnant with a specific emphasis on the type of medically assisted reproduction treatment used: fertility-enhancing drugs, intrauterine insemination (IUI), assisted reproductive technology (ART) with autologous or donor oocytes. We used high-quality data from Utah (2009–2017), a state with high proportion of medically assisted reproduction births (approximately 5%^4,20^) and compared maternal morbidity odds before and after adjustment for a wide range of characteristics, which might confound the association between maternal morbidity and medically assisted reproduction, such as pre-existing comorbidities, and for multifetal gestation and obstetric comorbidities, which might act as mediators.

## METHODS

We used data from the Utah Population Database,^21^ which contains information from all Utah birth certificates. The STROBE (Strengthening the Reporting of Observational Studies in Epidemiology) guidelines for cross-sectional studies were followed. Since 2009, Utah birth certificates contain data on infertility treatments used to become pregnant—fertility-enhancing drugs, IUI, and ART with autologous and donated oocytes (including donor embryos). We considered individuals reporting other treatments such as progesterone, metformin, and surgery for endometriosis as becoming pregnant unassisted (n=1,982), unless they also disclosed using one of the medically assisted reproduction procedures (n=5,134). At the time this study started, the Utah Population Database had received the birth certificate data up to 2017, marking the end of our study period. This study was approved by the institutional review boards of the University of Utah and by the Utah Resource for Genetic and Epidemiologic Research, an administrative board overseeing access to the Utah Population Database.

**Fig. 1.**
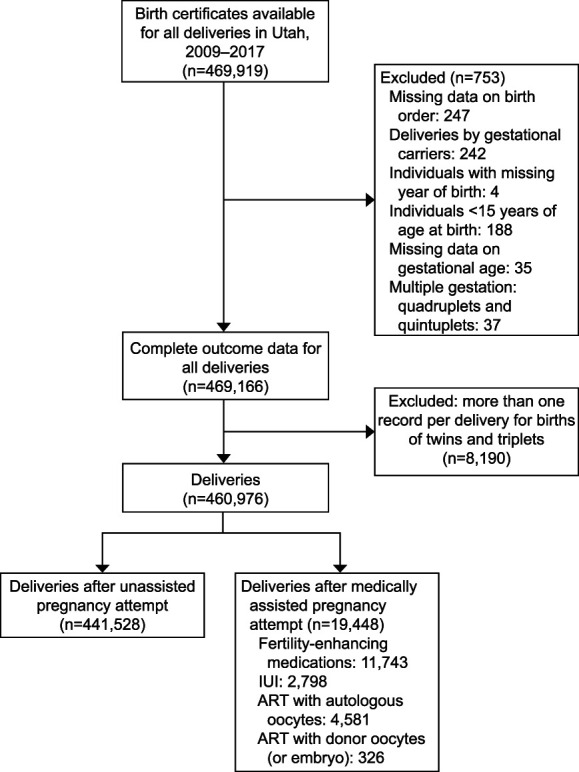
Study sample flow diagram. IUI, intrauterine insemination; ART, assisted reproductive technology.

The birth certificate data contains records for 469,919 deliveries registered in Utah. We excluded deliveries with missing birth order (n=247) and children born to gestational carriers (n=242). We also excluded quadruplet and quintuplet births (n=37). Further information on exclusions and missing data can be found in Figure 1. For twins and triplets, we considered one observation per delivery and controlled for multifetal gestation status. The final sample comprised 460,976 deliveries, of which 19,448 (4.2%) were medically assisted reproduction pregnancies.

*Pelikh. Maternal Morbidity and Medically Assisted Reproduction. Obstet Gynecol 2025*.

To identify maternal morbidity, we used all available information registered on the birth certificate under maternal morbidities: blood transfusion, unplanned operating room procedure, admission to intensive care unit, eclampsia, unplanned hysterectomy, and ruptured uterus. Though included under the maternal morbidities heading on the birth record, we did not include third- or fourth-degree perineal lacerations in our definition of maternal morbidity, because these are not included in other currently accepted and validated definitions.^22,23^ Maternal morbidity was coded as a binary variable, indicating the presence of any of the above events. This approach was chosen due to the low prevalence of individual maternal morbidity conditions when stratified by mode of becoming pregnant and medically assisted reproduction treatment type (as presented in Table 1, per 10,000 births), which could have compromised statistical precision if analyzed separately. Given the concerns on the accuracy of blood transfusion reporting and thresholds for consideration of severity of transfusion (number of units),^22,24^ we present analyses on the maternal morbidity composite score, including and excluding blood transfusion.

**Table 1.**
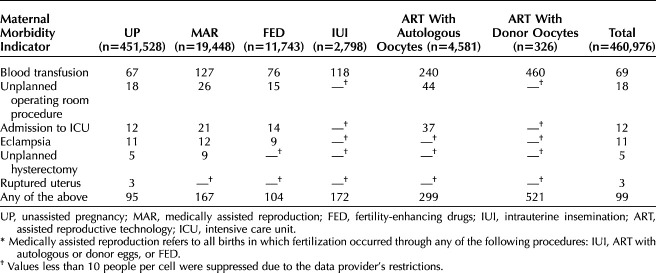
Rates of Maternal Morbidity Per 10,000 Births Among People Giving Birth in Utah, 2009–2017, by Mode of Becoming Pregnant and Type of Medically Assisted Reproduction Treatment*

We considered three sets of control variables (all coded as categorical variables). The first set of factors consisted of individual sociodemographic characteristics: age at birth (15–24 years, 25–29, 30–34, 35–39, 40–44, 45 or older), marital status, level of education (less than university degree, university degree or more) and parity (first or higher-order birth). We did not include race due to the very low proportion of Black, Asian, Pacific Islander, and Native American individuals who became pregnant through medically assisted reproduction in Utah (ie, fewer than 10 individuals for some race groups by treatment type), but we did include Hispanic origin. Collectively, these characteristics could confound the association between maternal morbidity and medically assisted reproduction.^25,26^

The next set of factors was related to health conditions and pre-existing comorbidities that could be associated with maternal morbidity and with experiencing subfertility.^14–17^ We incorporated data on asthma severity (severe and mild), chronic renal disease, chronic hypertension, heart disease severity (severe and mild), type 1 diabetes, and major mental health disorders (anxiety, depression, bipolar). We could not use the data on substance use, schizophrenia, rheumatic disease (rheumatoid arthritis, lupus, Sjogren's syndrome), and type 2 diabetes available on the birth certificate due to the very low prevalence of these conditions among individuals becoming pregnant through medically assisted reproduction. We included prepregnancy body mass index (BMI, calculated as weight in kilograms divided by height in meters squared; underweight [lower than 18.5], healthy weight [18.5–24.9], overweight [25.0–29.9], obesity [30–34.9, 35–39.9, 40 or higher]) given the demonstrated effects on both pregnancy complications and subsequent health, particularly among individuals undergoing medically assisted reproduction.^27^ We also accounted for smoking before pregnancy because it is a risk factor for adverse pregnancy outcomes.^28^ Additionally, we accounted for a history of prior caesarean deliveries, because it can influence subsequent mode of delivery and risk of maternal morbidity.^16^ We also accounted for multifetal gestation, which is a common risk factor for maternal morbidity.^29^

The last group of factors was linked to obstetric comorbidities that could be mediators (ie, conditions that develop in a pregnancy as a result of becoming pregnant through medically assisted reproduction) or confounders (ie, proxies for underlying health conditions before medically assisted reproduction [eg, subfertility]).^13–15,17^ Birth certificates contain information on the following conditions: placenta previa, placental abruption, preterm delivery, HELLP (hemolysis, elevated liver enzymes, and low platelet count) syndrome, pregnancy-induced hypertension, gestational diabetes, pyelonephritis, clinical chorioamnionitis, and delivery mode (cesarean delivery). Data on pyelonephritis were not included due to the very low prevalence among individuals conceiving through medically assisted reproduction. Information on hemorrhage was not included in the analysis because this condition is closely linked to blood transfusion.^13–15,17^

We estimated four multivariable logistic regression models for maternal morbidity. Model 1 (the baseline model) presents the unadjusted association between medically assisted reproduction and maternal morbidity. Model 2 introduces controls for sociodemographics, birth order, and pre-existing comorbidities. Model 3 adds multifetal gestation. Model 4 adds controls for obstetric comorbidities. Each model specification was estimated for all medically assisted reproduction treatments, grouped together and by treatment type, in comparison with becoming pregnant unassisted using the composite maternal morbidity score including and excluding blood transfusion as an outcome. We used clustered standard errors to account for multiple observations per woman (63.3% had one child only in the period 2009–2017). We first compared the prevalence of maternal morbidity among individuals who became pregnant through medically assisted reproduction and those who became pregnant unassisted using the whole sample. We then restricted the analysis to singletons only to examine the associations between maternal morbidity and medically assisted reproduction while removing the effects of multifetal gestation. Additionally, we estimated models that included an interaction between mode of becoming pregnant and pre-existing health conditions to explore whether they moderate the association between medically assisted reproduction and maternal morbidity. All analyses were conducted using STATA 18. *P*<.05 was used to denote statistically significant point estimates.

## RESULTS

Individuals who became pregnant through fertility-enhancing drugs were the largest medically assisted reproduction group (n=11,743; 60.4%, Fig. 1), followed by ART with autologous oocytes—23.5% (n=4,581), IUI—14.4% (n=2,798), and ART with donor oocytes—1.7% (n=326). Table 1 shows rates of all maternal morbidity per 10,000 births, by mode of becoming pregnant and treatments type (absolute numbers in Appendix 1, available online at http://links.lww.com/AOG/D939). Conditions are not mutually exclusive, so the total births with at least one condition is less than the sum of individual conditions. Blood transfusion was the most common maternal morbidity condition (69/10,000 births), followed by unplanned operating room procedure and admission to intensive care unit (18/10,000 births and 12/10,000 births, respectively). Maternal morbidity composite rates were higher among individuals who became pregnant through medically assisted reproduction, compared with unassisted reproduction (167/10,000 births vs 95/10,000 births, respectively). Within the medically assisted reproduction group, maternal morbidity rates varied by treatment invasiveness and were highest in the ART group (especially with donor oocytes—521/10,000 births vs 299 in autologous oocytes/10,000 births) and lowest in the fertility-enhancing drug group (104/10,000 births).

The sociodemographic and health characteristics of individuals who became pregnant through medically assisted reproduction differed from those who became pregnant without medically assisted reproduction (Table 2). Individuals becoming pregnant through medically assisted reproduction were, on average, two and a half years older at birth, more likely to have a degree, be married, and less likely to be of Hispanic origin. Their children were more likely to be first-borns and multifetal gestations (twins and triplets). Individuals conceiving through medically assisted reproduction also had higher rates of chronic hypertension and obesity but were less likely to smoke before pregnancy. The prevalence of other pre-existing comorbidities was similar among individuals who became pregnant through medically assisted reproduction and those who became pregnant unassisted (with some exceptions, eg, higher rates of asthma and major mental health disorders among individuals conceiving through ART with donor oocytes). Individuals who became pregnant through medically assisted reproduction had a higher prevalence of obstetric comorbidities regardless of treatment type compared with individuals who became pregnant unassisted.

**Table 2.**
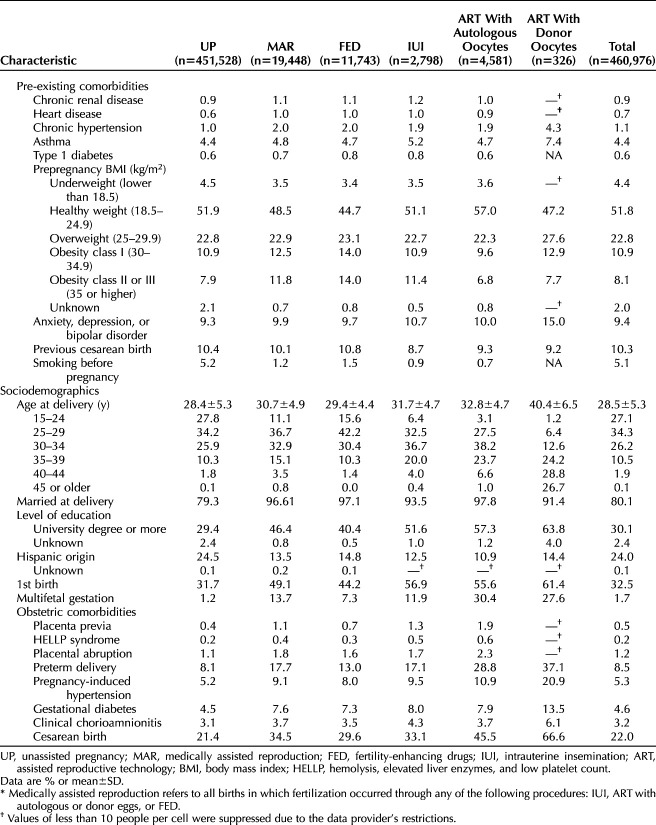
Individual and Pregnancy Characteristics and Obstetric Comorbidities Among People Giving Birth in Utah, 2009–2017, by Mode of Becoming Pregnant and Type of Medically Assisted Reproduction Treatment*

Table 3 shows medically assisted reproduction coefficients for maternal morbidity composite including and excluding blood transfusion (Panels A and B, respectively) from an unadjusted model (model 1), after adjusting for sociodemographic characteristics and pre-existing comorbidities (model 2), multifetal gestation (model 3) and obstetric comorbidities (model 4). Control variables coefficients for models 2–4 are in Appendices 2, 3 (singleton births), and 4 (all births), available online at http://links.lww.com/AOG/D939. In model 1, medically assisted reproduction was associated with higher odds of maternal morbidity, with odds ratios (ORs) being the highest among more invasive treatments (ART and IUI) and no differences between fertility-enhancing drugs and unassisted pregnancies. After adjusting for sociodemographics and comorbidities (model 2), the medically assisted reproduction–maternal morbidity association remained significant (OR 1.63, 95% CI, 1.45–1.84 including blood transfusion, and OR 1.29, 95% CI, 1.06–1.56 excluding blood transfusion). Age and parity were significant predictors of maternal morbidity in model 2 and in subsequent models. Adjusting for multifetal gestation (model 3) further reduced the OR, in particular among people who became pregnant through ART who have the highest prevalence of these pregnancies. In the models including blood transfusion, the odds remained higher and statistically significant for all medically assisted reproduction types compared with unassisted pregnancies, except for fertility-enhancing drugs. In the models that excluded blood transfusion, the OR remained significant only for ART pregnancies with autologous oocytes. Finally, adjusting for obstetric comorbidities (model 4) further attenuated the odds for all medically assisted reproduction types, but the OR remained significantly higher for ART with autologous oocytes (OR 1.46, 95% CI, 1.20–1.78) compared with unassisted pregnancies when blood transfusion was part of the score. However, as evident from Appendix 1 (http://links.lww.com/AOG/D939), the analyses excluding blood transfusion are likely to be underpowered, in particular when disaggregated by type of treatments. We are therefore cautious in our interpretation of the differences between the models.

**Table 3.**
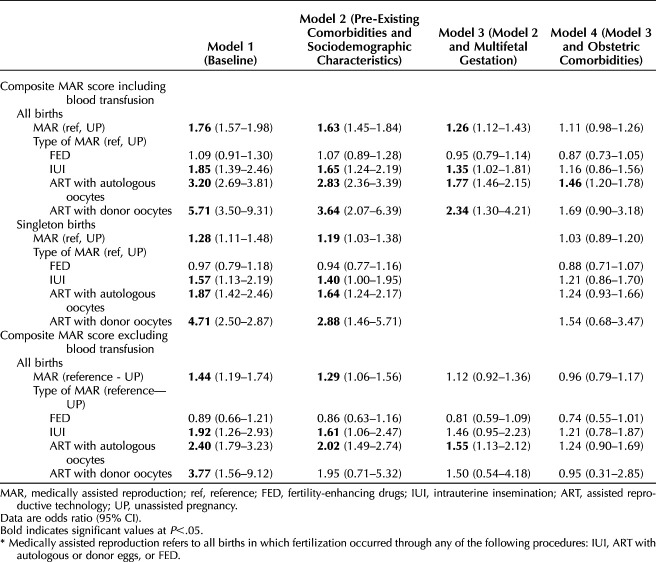
Maternal Morbidity Among People Giving Birth in Utah, 2009–2017: Medically Assisted Reproduction Compared With Unassisted Pregnancies*

In models including only singletons, the OR of maternal morbidity among all individuals who became pregnant through medically assisted reproduction (OR 1.28, 95% CI, 1.11–1.48) and by each treatment type was significantly higher compared with individuals who became pregnant unassisted (expect fertility-enhancing drugs), but the but the magnitude of the coefficients was, on average, lower compared with the coefficients in all-births model (Table 3) (Appendix 3, http://links.lww.com/AOG/D939). Similar to the full-sample results, associations varied by treatment type, with more invasive treatments linked to higher maternal morbidity risk. Adjustment for pre-existing health conditions and sociodemographic characteristics attenuated the coefficients, yet the OR of maternal morbidity remained significantly higher among all types of medically assisted reproduction (expect fertility-enhancing drugs). After controlling for obstetric comorbidities, the association between medically assisted reproduction and maternal morbidity was largely attenuated; the OR differences in maternal morbidity between all medically assisted reproduction groups and the unassisted pregnancy group were no longer statistically significant.

To investigate the moderating role of pre-existing comorbidities in the medically assisted reproduction–maternal morbidity association, we included an interaction term between mode of becoming pregnant and pre-existing health conditions (Table 4) (Appendix 5, http://links.lww.com/AOG/D939). Due to the lower prevalence of maternal morbidity by type of medically assisted reproduction treatment in the subgroups, we focused on fertility-enhancing drugs and ART pregnancies with autologous oocytes. In model 1 (unadjusted), ORs of maternal morbidity were higher among individuals with pre-existing comorbidities than those without comorbidities, regardless of the mode of becoming pregnant. However, individuals becoming pregnant through medically assisted reproduction without comorbidities had a higher OR of maternal morbidity than those who became pregnant without medically assisted reproduction with comorbidities. A similar pattern was observed for ART pregnancies. Adjustment for covariates in models 2–4 attenuated but did not eliminate these differences—individuals without comorbidities who became pregnant through ART with autologous oocytes had significantly higher ORs of maternal morbidity, whereas no differences in OR were found among individuals conceiving through fertility-enhancing drugs.

**Table 4.**
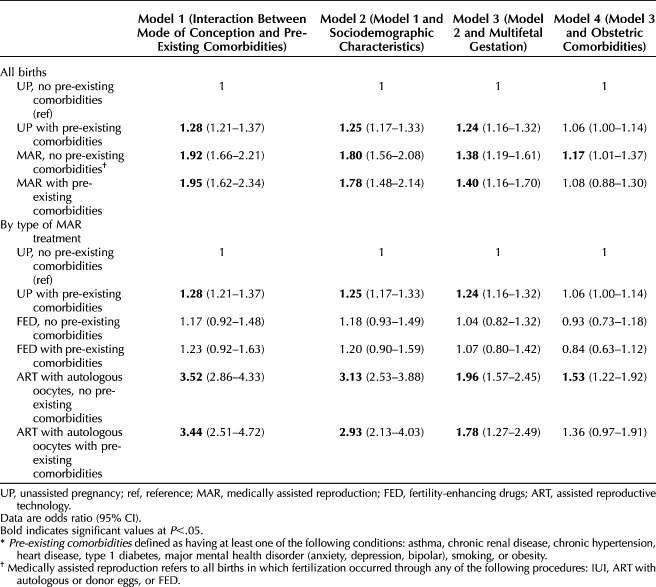
Maternal Morbidity Among People Giving Birth in Utah, 2009–2017, by Mode of Becoming Pregnant and Pre-Existing Comorbidities*

## DISCUSSION

This study examines the association between various medically assisted reproduction treatments and maternal morbidity in Utah, using birth certificate data. Results reveal that multifetal gestations significantly raise maternal morbidity risk among individuals who become pregnant through medically assisted reproduction, aligning with existing research.^29^ Notably, maternal morbidity risk for singleton births remains lower, underscoring the contribution of multifetal pregnancies to maternal morbidity in medically assisted reproduction cases. Sociodemographic factors such as age and parity also play a role; many medically assisted reproduction children are first-born, and nulliparity is a known maternal morbidity risk. Age-related differences in medically assisted reproduction outcomes were especially pronounced in ART pregnancies involving donor oocytes, with older average maternal age (40.4 years) compared with non-ART births (mean age 28.4 years and 30.7 years among individuals with unassisted pregnancy and medically assisted reproduction-all, respectively).

Prior studies^14–17^ suggest that the higher rates of pre-existing comorbidities among individuals who became pregnant through medically assisted reproduction might also play a role. The results showed that, although pre-existing comorbidities moderated the association between maternal morbidity and medically assisted reproduction, medically assisted reproduction pregnancies remained at higher risk of maternal morbidity even if they did not have pre-existing comorbidities, which suggests they only play a partial role in explaining the association. Another potential mechanism linking medically assisted reproduction and maternal morbidity is the increased rate of obstetric complications, such as placenta previa, that occur in medically assisted reproduction pregnancies. Accounting for these, the medically assisted reproduction–maternal morbidity relationship remained significant among ART conceptions with autologous oocytes. However, it is unclear whether obstetric comorbidities are caused by the treatments, as the higher prevalence of obstetric risk factors among ART conceptions could be driven by subfertility and not by the treatments.

The study's strengths include using Utah's high-quality, population-wide vital records, which enable a large sample size for medically assisted reproduction pregnancies. Although underreporting of ART use on birth certificates is a known issue,^30^ data quality in Utah is comparatively reliable,^31^ minimizing this concern. This study also distinguishes specific medically assisted reproduction treatments in relation to maternal morbidity and accounts for a wide range of factors such as sociodemographics, maternal comorbidities, and pregnancy characteristics. Comparison with medical records shows high reporting quality for relevant perinatal outcomes, enhancing the study's validity.^32–34^

However, limitations remain. The birth certificates used lack certain recommended health indicators (eg, preeclampsia), and low reporting rates for some comorbidities may affect multivariable model accuracy. Additionally, the data do not differentiate specific ART protocols or monozygotic from dizygotic multifetal gestations, which are important given their distinct maternal morbidity risks.^1,4–15,35,36^ The absence of comprehensive histories for fertility treatments or detailed subfertility factors (eg, infertility duration or underlying causes) limit exploration of subfertility's role in maternal morbidity outcomes.

This study adds to existing knowledge by showing a dose-response relationship where maternal morbidity risk rises with treatment invasiveness, consistent with past findings of higher maternal morbidity in ART pregnancies.^5–12^ Notably, unlike previous studies, this research differentiates fertility-enhancing drugs from IUI and shows similar maternal morbidity odds between fertility-enhancing drugs and unassisted pregnancies. Assisted reproductive technology with donor oocytes had the highest maternal morbidity odds, likely linked to older maternal age, multifetal gestation, and treatment invasiveness.

This study also contributes to the discussion on the role of subfertility and medically assisted reproduction procedures in explaining the increased maternal morbidity among medically assisted reproduction pregnancies.^5,6,12^ Our findings showing that the odds of maternal morbidity increase with more invasive treatments suggest that subfertility could be an important underlying factor as it is associated with both more invasive medically assisted reproduction procedures and maternal morbidity. Nonetheless, they could also point to the role of the medically assisted reproduction procedure themselves. Invasive medically assisted reproduction procedures are likely on the pathway between subfertility and maternal morbidity, and different types of more invasive ART-associated procedures (eg, ICSI, embryo biopsy) may play a greater or lesser mediating role in the relationship. Unfortunately, our data do not allow us to elucidate these more granular relationships, though we did account for many obstetric comorbidities (eg, multifetal gestation and placenta previa), which could be associated with procedures such as embryo biopsy. Alternatively, there may also be less well understood contributors to the pathophysiology of more severe or intractable subfertility or infertility itself (eg, immune milieu, microvascular dysfunction, chronic inflammation) that could independently predispose individuals needing medically assisted reproduction to experience maternal morbidity. More research is needed to further test these associations.

The findings have implications for patients, clinicians, and public health policymakers. Increased maternal morbidity risk in medically assisted reproduction-related multifetal pregnancies highlights potential adverse health effects and costs, suggesting that minimizing multifetal gestation is crucial.^37,38^ Multiple pregnancies are often linked to specific fertility drug protocols or multiple embryo transfers during ART to improve pregnancy odds with fewer treatment cycles.^39,40^ Nordic countries have seen decreased multiple births in ART due to elective single embryo transfer (eSET), an approach the United States is gradually adopting (rates rose from 7% in 2009–67.3% in 2017 for individuals younger than 35).^41,42^ However, the lack of state-funded provision and high treatment costs may encourage multiple embryo transfers in the United States, emphasizing the need for awareness campaigns on eSET's benefits, which can offer comparable pregnancy success rates while reducing maternal morbidity risks.^43,44^ Public health initiatives promoting eSET and counseling on maternal morbidity risks associated with medically assisted reproduction-related multifetal pregnancies can guide safer treatment choices, potentially lowering maternal morbidity rates and related costs.
